# Comparison and validation of accelerometer wear time and non-wear time algorithms for assessing physical activity levels in children and adolescents

**DOI:** 10.1186/s12874-019-0712-1

**Published:** 2019-04-02

**Authors:** Jérémy Vanhelst, Florian Vidal, Elodie Drumez, Laurent Béghin, Jean-Benoît Baudelet, Stéphanie Coopman, Frédéric Gottrand

**Affiliations:** 1 University of Lille, Inserm, CHU Lille, U995 – LIRIC – Lille Inflammation Research International Center, CIC 1403 – Centre d’investigation clinique, Avenue Eugène Avinée, 59037 Lille Cedex, F-59000 Lille, France; 2 University of Lille, CHU Lille, EA 2694 – Public Health: epidemiology and quality of care, F-59000 Lille, France

**Keywords:** Young, Activity monitor, Free living conditions, Wear time, Algorithms

## Abstract

**Background:**

Accelerometers are widely used to measure sedentary time and daily physical activity (PA). However, data collection and processing criteria, such as non-wear time rules might affect the assessment of total PA and sedentary time and the associations with health variables. The study aimed to investigate whether the choice of different non-wear time definitions would affect the outcomes of PA levels in youth.

**Methods:**

Seventy-seven healthy youngsters (44 boys), aged 10–17 years, wore an accelerometer and kept a non-wear log diary during 4 consecutives days. We compared 7 published algorithms (10, 15, 20, 30, 60 min of continuous zeros, Choi, and Troiano algorithms). Agreements of each algorithm with the log diary method were assessed using Bland-Altmans plots and by calculating the concordance correlation coefficient for repeated measures.

**Results:**

Variations in time spent in sedentary and moderate to vigorous PA (MVPA) were 30 and 3.7%. Compared with the log diary method, greater discrepancies were found for the algorithm 10 min (*p* < 0.001). For the time assessed in sedentary, the agreement with diary was excellent for the 4 algorithms (Choi, *r* = 0.79; Troiano, *r* = 0.81; 30 min, *r* = 0.79; 60 min, *r* = 0.81). Concordance for each method was excellent for the assessment of time spent in MVPA (> 0.86). The agreement for the wear time assessment was excellent for 5 algorithms (Choi r = 0.79; Troiano *r* = 0.79; 20 min *r* = 0.77; 30 min *r* = 0.80; 60 min *r* = 0.80).

**Conclusions:**

The choice of non-wear time rules may considerably affect the sedentary time assessment in youth. Using of appropriate data reduction decision in youth is needed to limit differences in associations between health outcomes and sedentary behaviors and may improve comparability for future studies. Based on our results, we recommend the use of the algorithm of 30 min of continuous zeros for defining non-wear time to improve the accuracy in assessing PA levels in youth.

**Trial registration:**

NCT02844101 (retrospectively registered at July 13th 2016).

**Electronic supplementary material:**

The online version of this article (10.1186/s12874-019-0712-1) contains supplementary material, which is available to authorized users.

## Background

Physical activity (PA), especially moderate-to-vigorous PA (MVPA), positively influences health in children and adolescents [1, 2]. By contrast, a sedentary lifestyle is associated with adverse health consequences such as obesity, lower aerobic fitness, poorer quality of life and self-esteem, depression, and anxiety [3]. The accurate measurement of PA levels is essential for evaluating the relationships between PA and health outcomes in both epidemiological and interventional studies.

The use of accelerometers has become the method of choice for objectively assessing PA levels [4]. However, this method has some limitations for data collection and processing criteria decisions. One major concern is the validation of wear and non-wear time when the accelerometer is worn at the hip. Participants are usually instructed to remove the device during water-based activities (swimming, showering, and bathing) and overnight. This requires the use of a non-wear log diary to determine whether the participant has worn the device during a sedentary activity or removed the device (and therefore PA would be missed), but this is a cumbersome procedure that can create barriers in large population studies.

To solve this issue, two methodologies have been used. Firstly, researchers can to perform their assessment using wrist-worn accelerometers. Indeed, wrist-worn accelerometers, compared to waist-worn monitors, may be more convenient and comfortable and improve compliance in studies where there is prolonged wear time (usually 7 day to assess habitual physical activity) [5]. As second possibility, algorithms have been proposed to consider the treatment of consecutive zeros recorded by the accelerometer as an indicator of non-wear times when participant wear at hip. Several cutoffs are used as an indicator of a non-wearing period, such as 10, 15, 20, 30, or 60 min of continuous zeros [6–12]. Other algorithms that consider additional parameters have been also proposed [11, 13]. However, the use of these algorithms carries a risk of falsely classifying a true sedentary period as a non-wear period, and the choice of the best algorithm remains a matter of debate [13–24]. Few studies have addressed this question in youth [20–24]. These studies showed clearly that the choice of wear time algorithms may introduce significant errors in PA levels assessment [20–24]. In a review, Esliger et al. suggested to use 20 min of consecutive zero counts as criterion in children [24]. This conclusion concurs with another study performed in 369,517 children aged 8–13 years [23]. Authors compared three non-wear time algorithms (10, 20 and 60 min of consecutives zero counts) with a data reduction log [23], and found that 20 min is the more appropriate. Chinapaw et al. suggested that the 20 min algorithm was to low and recommended a minimum of 60 min of consecutive zeros as the most realistic criterion for non-wear time [22]. Based on 268 children aged 7–11 years, Banda et al. compared other algorithms (20 min, Choi and Troiano algorithms) and showed also a significant error in PA assessment [20]. However, authors compared different non-wear time definitions without a non-wear log diary. Therefore, authors could not recommend the better algorithm for data processing reduction. Recently, a study compared 10 algorithms with a logbook in children aged 10 years [21]. Authors concluded a 45–60 min criterion being used in future studies [21]. However, in this study, no specific time points for wear and non-wear were provided in the logbook that could lead to an error in the ability to identify the better algorithm [21]. Indeed, to be more precise and for determining the exact agreement for each period as wear and non-wear and the accelerometer files, only a comparison with total wear time is not enough robust. Using data from the diary with specific time points for wear and non-wear would provide a better basis for evaluating the performance of the non-wear time criteria and would provide new knowledge in the field. These conflicting results between all these studies lead confusion for researchers and practitioners who have to make better decisions in their data collection using accelerometer, in order to obtain more valid and comparable data.

Therefore, the aim of this study was to investigate whether the choice of different non-wear time definitions would affect the measurement of PA levels in children and adolescents. The final aim of this study was to identify the best algorithm for assessing non-wear time using a log diary as the reference method.

## Methods

### Participants

Eighty healthy children and adolescents participated in this study. The inclusion criteria were: (*i*) boys and girls aged 10–18 years; (*ii*) informed consent form signed by the participant and parents; (*iii*) no medical contraindication against daily practice of PA; and (*iv*) no simultaneous participation in another biomedical study. Participants who did not record at least 3 days of recording (according to the log book) were excluded from the analyses.

All parents/guardians signed an informed consent form, and the adolescents agreed to participate in the study. The study was performed following the ethical guidelines of the 1964 Declaration of Helsinki (revision in 2008), the Good Clinical Practice, and legislation regarding clinical research in humans. The study was approved by the Human Research Review Committee (Comité Protection des Personnes, Nord Ouest IV, Lille, France).

### Procedures

On the test day, body mass without shoes or heavy outer garments was measured to the nearest 0.1 kg using an electronic scale (Seca, Hamburg, Germany). Height without shoes was measured to the nearest 0.1 cm using a stadiometer (Seca). Participants were asked to wear the accelerometer during 4 consecutive days (2 school days and 2 school-free days) on their lower back under their clothing using an elastic belt and adjustable buckle. They were asked also to follow their normal daily routine. They were instructed to remove the device before contact sports, swimming, showering, or bathing and at night before sleep. Participants also wore a watch (Vivago; Vivago Wellness, Paris, France) that was initialized and synchronized with the accelerometer by the same computer (i.e., time setup: local computer time for both the accelerometer and watch). Participants were also instructed to keep a log diary while wearing the accelerometer.

### Measurements

#### Accelerometer

The activity monitor used for this study was the triaxial ActiGraph accelerometer (Model GT3X; ActiGraph, Pensacola, CA, USA). This small, lightweight triaxial accelerometer (46 × 33 × 15 mm; 19 g) has been validated for assessing physical activity and shows a high reliability [25, 26]. This device assesses PA by measuring mechanical movement in the three dimensions of space: (*i*) vertical vector (x), (*ii*) an anteroposterior vector (y), (*iii*) and a mediolateral vector (z). Vector magnitude is calculated as the square root of the sum of squared activity counts for each vector.

ActiGraph software support (ActiLife, v6.13.2, Pensacola, CA, USA**)** was used to initiate, download and process data. The accelerometers were calibrated according to the age, height, and weight of the user. The epoch interval for the accelerometer was set at 1 s, and the output was expressed as counts per min. The same computer was used for the initialization and synchronization of the accelerometers. Data were uploaded from the monitor to a computer after the completed 4-day registration period. Physical activity levels were divided into the following classifications: sedentary activity, 0–180 counts.15 s^− 1^; light activity, 181–757 counts.15 s^− 1^; moderate activity, 758–1112 counts.15 s^− 1^; and vigorous activity, > 1112 counts.15 s^− 1^ [27].

#### Non-wear log diary

The participants were asked to keep a log diary during the 4-day period when they wore the accelerometer (See Additional file 1). The times of waking and going to bed, and the times when the accelerometers were put on and taken off were recorded daily on a standardized, preprinted recording sheet. To ensure the accuracy of the assessment of non-wear time, the participants were asked to report in the log diary the exact time (hour and min) using the watch provided specifically for the study, which had been synchronized with the accelerometer. In addition, the participants completed the log diary in real time. We calculated the non-wear time for each day by summing the duration noted on the log diary until the accelerometer was removed at night or for others activities.

#### Non-wear time from accelerometer

We applied 7 different algorithms to calculate non-wear time from the accelerometer records using ActiGraph software support (ActiLife, v6.13.2, Pensacola, CA, USA**)**. Five algorithms consider 10, 15, 20, 30, and 60 min of continuous zeros to indicate a non-wear period. The Troiano algorithm (2008) uses a minimum of 60 min of 0 counts per min with an allowance of 2 min of interruptions, whereas the algorithm developed by Choi et al. (2012) considers a minimum of 90 min of 0 counts per min with an allowance of 2 min of interruptions plus two 30 min windows of 0 counts per min before and after that allowance [11, 15]. We compared PA levels obtained after applying these 7 algorithms with those calculated using the non-wear log diary as the reference method.

#### Valid days

A minimum of 10 h of wearing time is needed to consider the day as valid in the assessment of PA in youth using accelerometry [28]. We compared the percentage of participants meeting the recommendation of a minimum of 10 h wearing time according to the log diary and different algorithms.

### Statistical analysis

Comparison of log diary method (considered as gold standard) to each other algorithm methods for the measurement of wear time and PA levels were done used linear mixed models with random intercept effect to take into account multiple measurements per participant; in these models, algorithm methods and time were included as fixed effects. Agreements of each algorithm with the gold standard method (log diary method) were assessed using Bland Altman’s plots and by calculating the concordance correlation coefficient (CCC) for repeated measures to take into account the 4 days recorded per participant [29, 30]. CCC values were interpreted as: low concordance of values < 0.45, reasonably good 0.45–0.75, and excellent > 0.75 [31]. We also calculated Kappa coefficient for agreement between algorithm methods to classified days where adolescents fulfilling the recommendations of 60 min of MVPA. The chi-square test was used to compare the percentages of participants meeting the recommendation of a minimum of 10 h wearing time with those calculated using the gold standard method (log diary method). Weighted Cohen’s Kappa was calculated for agreement between algorithm methods on the number of non-wear period, classified as 0, 1, 2, 3, 4 and 5 or more.

All statistical tests were performed at the 2-tailed α level of 0.05. Data were analyzed using SAS software (version 9.4; SAS Institute Inc., Cary, NC, USA) and R software (version 3.5.1).

## Results

Of the 80 participants who wore an accelerometer throughout the 4 days, 3 were excluded because of monitoring failure (*n* = 3). The physical characteristics of the 77 participants included in the study are described in Table 1.

**Table 1:** Physical characteristics of the participants (mean ± SD)

Boys/girls (*n*)	44/33
Age (*years*)	13.2 ± 2.2
Height (*cm*)	156.6 ± 13.6
Weight (*kg*)	46.4 ± 12.3

Wear times and PA levels grouped according to the algorithm used are presented in Table 2. Wear time varied by 17% according to the algorithm used. Compared with the log diary method, the discrepancy was largest (12%) for the algorithm using 10 min of continuous zeros (*p* < 0.0001).

**Table 2:** Wear time and physical activity levels (min ± SD) according to the algorithm used

	Wear time (*min.day*^*− 1*^)	P*	Levels of PA (*min.day*^*− 1*^)			
			Sedentary	P*	MVPA	P*
Log diary method	727.5 ± 154.2	–	515.2 ± 134.1	–	60.9 ± 42.9	–
Troiano et al. (2008)[11]	756.0 ± 140.4	0.006	541.0 ± 121.1	0.002	58.6 ± 38.3	0.38
Choi et al. (2012)[15]	767.0 ± 137.9	0.0001	551.0 ± 125.9	< 0.0001	58.8 ± 38.3	0.42
60 min	764.4 ± 135.0	0.0003	548.3 ± 122.7	< 0.0001	58.8 ± 38.3	0.42
30 min	736.4 ± 139.5	0.38	520.4 ± 118.1	0.54	58.8 ± 38.3	0.42
20 min	712.2 ± 144.1	0.14	496.2 ± 114.2	0.026	58.8 ± 38.3	0.42
15 min	689.1 ± 148.7	0.0003	473.1 ± 111.7	< 0.0001	58.8 ± 38.3	0.42
10 min	640.4 ± 154.6	< 0.0001	424.2 ± 108.1	< 0.0001	58.8 ± 38.3	0.42

*P-value calculated using a linear mixed model using the gold standard method (log diary method) as reference

Time spent in sedentary PA varied by 30% according to the algorithm used. Compared with the log diary method, the discrepancy was largest (18%) for the algorithm using 10 min of continuous zeros (*p* < 0.001), but there was no significant difference between the log diary method and the algorithm 30 min. Differences in time spent in MVPA calculated using all of the algorithms were small and not significant (3.5–3.7%) (*p* > 0.38).

Table 3 shows the agreement between the different algorithms used and the log diary method for the assessment of wear time and time spent in sedentary activities and MVPA. The agreement for the wear time assessment was excellent for 5 algorithms, i.e., (*i*) Choi, (*ii*) Troiano, (*iii*) 20 min, (*iv*) 30 min, and (*v*) 60 min; reasonably good for the algorithm 15 min; and low for the algorithm 10 min. For the time assessed in sedentary activities, the agreement with the gold standard was excellent for 4 algorithms, i.e., (*i*) Choi, (*ii*) Troiano, (*iii*) 30 min, (*iv*) and 60 min; reasonably good for 2 others: (*i*) 15 min, (*ii*) and 20 min; and low for 1 algorithm (10 min). Concordance for each method was excellent for the assessment of time spent in MVPA (CCC > 0.86).

**Table 3:** Concordance correlation coefficients between algorithms with the log diary method

	Wear time	Sedentary time	MVPA time
Troiano et al. (2008)[11]	0.79 [0.65; 0.88]	0.81 [0.72; 0.87]	0.86 [0.81; 0.90]
Choi et al. (2012)[15]	0.79 [0.66; 0.88]	0.79 [0.70; 0.86]	0.86 [0.80; 0.90]
60 min	0.80 [0.67; 0.89]	0.81 [0.72; 0.87]	0.86 [0.81; 0.90]
30 min	0.80 [0.66; 0.88]	0.79 [0.69; 0.86]	0.86 [0.81; 0.90]
20 min	0.77 [0.63; 0.86]	0.73 [0.61; 0.82]	0.86 [0.81; 0.90]
15 min	0.73 [0.57; 0.83]	0.65 [0.51; 0.76]	0.86 [0.81; 0.90]
10 min	0.60 [0.43; 0.73]	0.46 [0.31; 0.59]	0.86 [0.81; 0.90]

Concordance correlation coefficients and theirs 95% confidence intervals for the agreement assessment between methods with the gold standard method (log diary method)

For the estimation of the number of days in which the participants met the recommendation of 60 min of MVPA per day, there was an excellent concordance for each algorithm with the log diary method (Table 4). The number of days when adolescents met the recommendation of 60 min of MVPA per day did not differ between the log diary and algorithms (Table 4).

**Table 4:** Number of days where adolescents fulfilling the recommendations of 60 min of MVPA per day

	Days (*n*)	Kappa Coefficient [95% confidence intervals]^a^
Log diary method	179	
Troiano et al. (2008)[11]	178	0.88 [0.84; 0.91]
Choi et al. (2012)[15]	179	0.89 [0.86; 0.91]
60 min	178	0.89 [0.86; 091]
30 min	178	0.89 [0.86; 091]
20 min	178	0.89 [0.86; 091]
15 min	178	0.89 [0.86; 091]
10 min	178	0.89 [0.86; 091]

^a^Kappa coefficient for the agreement assessment between methods with the gold standard method (log diary method)

**Fig. 1:**
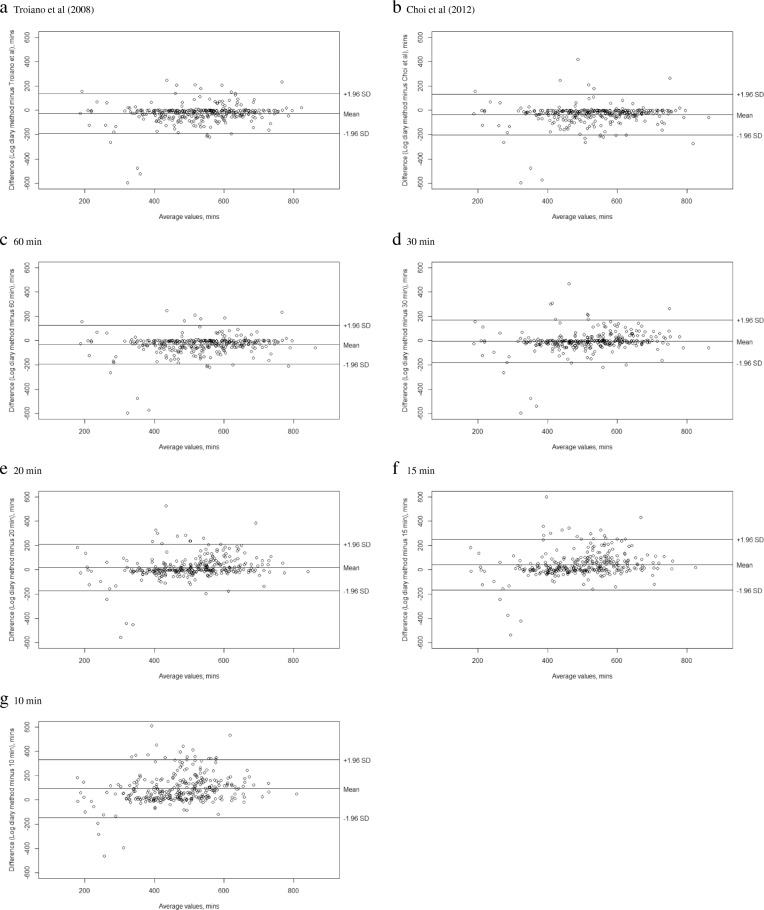
Bland and Altman plots for concordance between algorithms with the log diary method in the assessment of time spent in sedentary activities (**a** Troiano algorithm, **b** Choi algorithm, **c** 60 min algorithm, **d** 30 min algorithm, **e** 20 min algorithm, **f** 15 min algorithm, **g** 10 min algorithm)

Bland and Altman plots for concordance between algorithms with the log diary method in the assessment of time spent in sedentary activities are presented in Fig. 1. The mean error of bias varied between − 5.2 min.day^− 1^ and 90.9 min.day^− 1^. The lowest and highest levels of agreement were found for 10 min algorithm and 30 min algorithm, respectively.

**Fig. 2:**
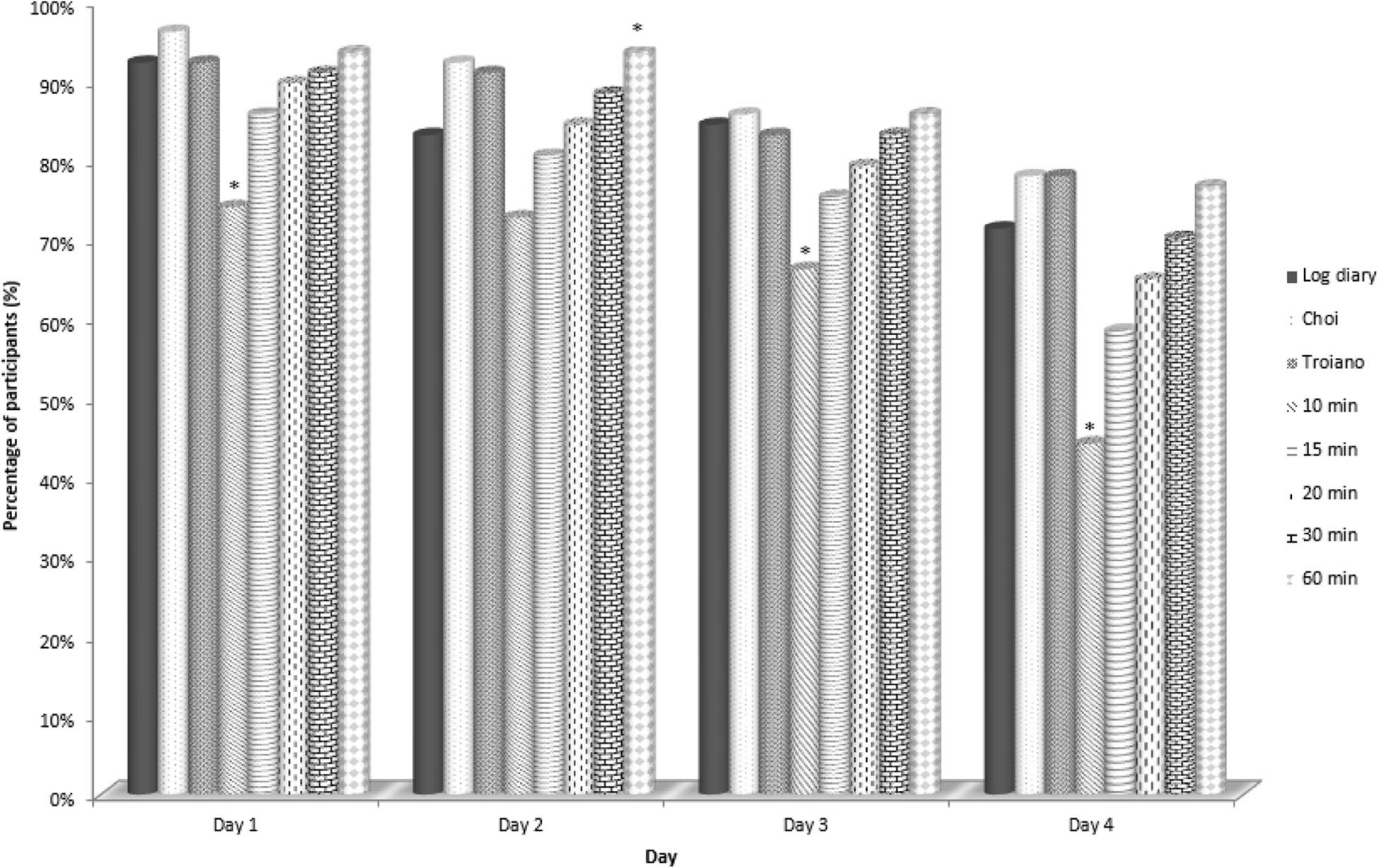
Percentage of participants meeting the recommendation of minimum of 10 h wearing time according to algorithms used

Figure 2 shows the percentages of participants meeting the recommendation of a minimum of 10 h wearing time (i.e., a valid day) according to the algorithms used. The algorithms of 10 min and 60 min differed significantly compared with the log diary (*p* < 0.05).

Number of non-wear periods per day according to log diary and the different non-wear criteria are presented in Table 5. Number of non-wear periods per day as determined by accelerometry ranged from 0 to 25, as opposed to 0–3 for the log diary. Lengthening of non-wear time duration has an impact on the number of non-wear periods. The algorithm of 10 min assessed the maximum number of periods during the day to 25 while the algorithm of 60 min detected a maximum of 3 non-wear periods. When comparing the mean of the number of non-wear periods between log diary method and different non-wear time criteria, using the algorithm of 30 min seems to be more adequate (mean difference of 0.20).

**Table 5:** Distribution of the number of non-wear periods according to the algorithm used

	Frequency (% of days)						Range	Kappa^a^
	0	1	2	3	4	≥ 5		
Log diary method	50.7	42.5	5.2	1.6	0	0	0–3	–
Troiano et al. (2008)[11]	77.3	17.9	4.5	0.3	0	0	0–3	0.11 [0.02; 0.20]
Choi et al. (2012)[15]	90.1	8.5	1.4	0	0	0	0–2	0.09 [0.02; 0.16]
60 min	70.1	25.0	4.2	0.7	0	0	0–3	0.09 [−0.01; 0.18]
30 min	47.4	32.5	16.9	2.3	0.3	0.6	0–5	0.17 [0.08; 0.25]
20 min	26.6	29.6	20.1	10.7	4.9	8.1	0–11	0.07 [0.03; 0.12]
15 min	2.3	4.2	9.1	12.4	9.7	62.3	0–25	0.01 [0.00; 0.02]
10 min	1.9	3.3	9.7	10.1	11.4	63.6	0–25	0.01 [0.00; 0.01]

^a^Weighted Cohen’s Kappa coefficients and theirs 95% confidence intervals for the agreement assessment between methods with the gold standard method (log diary method)

## Discussion

In a review of accelerometer methods and decision rules for PA measurement in children, Cain et al. showed that non-wear time was the most frequent parameter missing in 43–65% of published studies [32]. Our study clearly shows that wear time can vary widely according to the algorithm used and that this variability significantly affects the assessment of sedentary activity but not MVPA. Therefore, defining the most accurate method for assessing non-wear time is needed to standardize PA measurement using accelerometry in youth.

One main result of our study was that the cutoff for non-wear time of an algorithm strongly affects the assessment of the sedentary activity duration. This result is in agreement with previous studies performed in children, adults, and elderly people [14, 16–23]. Toftager et al. showed a difference of 10% in the assessment of sedentary behaviors of children between the 90 min and 10 min non-wear time algorithms [14]. Janssen et al. found a difference of 17% in the mean daily sedentary time using 10 and 60 min rule [23]. Two others studies in children demonstrated also that time spent in sedentary activity varied significantly according to wear time algorithms [20, 22]. However, because authors compared different non-wear time definitions without a non wear log diary, their reported analyses were insufficiently consistent to be able to identify a better algorithm for data processing reduction for assessing sedentary behaviors. Recently, a study compared 10 algorithms with a logbook in children and authors encouraged to use a 45–60 min criterion being used in future pediatric studies for assessing sedentary time [21]. In our study, 3 algorithms (Choi, Troiano, and 60 min of continuous zeros) significantly overestimated the time spent in sedentary activities and 3 others algorithms (10, 15, and 20 min of continuous zeros) significantly underestimated the time spent in sedentary activities. The best agreement was found using the criterion of 30 min of continuous zeros, which differed by only 1% difference from the log diary. Methodological issues might explain the discrepancy we found in the present study. In our study, we used specific time points for wear and non-wear provided in the diary. Our dataset allows for determining the exact agreement for each period as wear and non-wear from the diary and the accelerometer files, not only a comparison of total wear time in the Aadland et al’s study. The wide range of ages used in our study may also explain the difference. We analysed data from children and adolescents while Aadland et al. were included only children.

The number of non-wear periods according to log diary ranged 0 to 3 periods per day. Our results are in agreement with those found by Aadland et al. where authors reported 0–3 non wear periods per day using a log [21]. As concluded previously also, it would seem unlikely that children remove the accelerometer more than 3–4 times during one assessment day [21, 22]. This conclusion concurs with our current findings showing the best agreement between log diary and assessment of the sedentary activity duration for the criterion of 30 min of continuous zeros (Kappa coefficient = 0.17; 0–5 non-wear periods per day). While our study found a maximum of 3 non-wear periods for the 30 min non-wear algorithm, previous studies have found a maximum number of 5–7 periods for this algorithm, which supports the choice of a longer non-wear criteria [14, 21, 22]. Methodological issues in the study design and sample size differences might explain the discrepancies found in the present study [14, 21, 22]. In our study, the age range was more large (10–17 years) compared with previous studies (9–14 years). The difference in our results might be due also of a smaller sample (*n* = 77) compared to the previous studies having around 1000 participants. Then, sociodemographic characteristics and the length of assessment period (4 days vs 7 days) could also contribute in these conflicting results. Further studies are needed to analyze these differences in order to suggest the best algorithm for children and adolescent.

Another main result of our study was that the choice of algorithm had no significant effect on MVPA assessment and the percentage of participants meeting the PA recommendations. Only 1 study has examined this outcome in adults and it also found no significant difference in the percentage of participants classified as meeting the PA recommendations [17]. In the study performed in children by Aadland et al., authors found no difference in time spent in MVPA between algorithms [21]. Therefore, we can extrapolate that the number of children that achieved the guideline amount of MVPA according algorithms is obviously similar. Results from these studies show clearly that the choice of wear time algorithms do not impact in the MVPA assessment.

According to consensus recommendations for assessing PA in youth with accelerometers, a minimum of 10 h of wearing time is needed to consider the day as valid [28]. The algorithm comparisons in our study show that the choice of algorithm may significantly affect the duration of wear time and the choice whether to consider a recording day as a valid day. As for sedentary time assessment, the same algorithms under- or overestimated the wear time duration, expect the algorithm with 20 min or 30 min of continuous zeros. Using longer non-wear definitions (such as the Troiano and Choi algorithms or 60 min) resulted in overestimation of the wear time compared with the log diary. Our data differ from those of previous studies in older adults, in which longer non-wear definitions (i.e., at least 60 min) provided the closest approximation of self-reported wear time [15, 18]. These authors recommended using a longer interruption period when collecting accelerometer data from elderly people to maximize sample size and to provide the most accurate estimation of wear and sedentary time [15, 18].

Given the effects of different algorithms on the assessment of time spent in sedentary activities and wear time, and based on our findings, researchers should clearly specify the methods used for data collection and the processing criteria when estimating these parameters. Using the results of our study, we recommend the use of the algorithm with 30 min of continuous zeros for defining non-wear time for PA when using accelerometers in children and adolescents. This recommendation disagrees with that for adults and children; thresholds of 60 min and 20 min of consecutive zeros for adults and children are recommended to avoid the risk of misclassification of non-wear time as sedentary time [33]. The decline in PA with age is a possible explanation for the difference with our results [23, 34, 35]. Adults and older adults spend more time in sedentary activities than do children and adolescents. Therefore, using a too-short non-wear algorithm may increase the risk of misclassifying sedentary time as non-wear time in adults. Conversely, children spend less time in sedentary activities than adolescents. Indeed, authors showed significant differences in sedentary time between the different non wear rules from childhood to adolescence. Compared to the manual rule, at age 9 years, the 10 min zero string non wear rule resulted in the closest estimates of sedentary time while at age 12 years, the 20 min and 60 min zero-string rule are better, respectively [23]. Our recommendation is also support when we compared the mean of the number of non-wear periods between log diary method and different non-wear time criteria. Our finding suggests that use the algorithm of 30 min seems to be more adequate.

The current study has strengths and limitations. The main strength of the study is the use of a log diary as the basis for comparison of different non-wear times; this provides confidence in our findings. One limitation is the precision in the data recording in the log diary. However, participants used a specific watch that had been synchronized with the accelerometer to improve the accuracy of the assessment of non-wear-time. In addition, inclusion criterion was compliance of participants in completing this log diary. Even if several authors reported a bias in PA assessment with log diary in youth, our results are robust because the primary objective of our study was to use the log diary for assessing non-wear time and not their PA levels. A second limitation is our study is that it involved only healthy volunteers who were relatively active, which limits our results to this population. Overweight and obese adolescents spend more time in sedentary activities compared with their lean counterparts, which may increase the risk of misclassifying sedentary time as non-wear time in overweight and obese youths. Further studies should include a more heterogeneous population to confirm and extend our findings. The wide range of ages (10–17 years) used in our study may also have an impact on our results. Adolescents spending more time in sedentary activities compared to children [35], we cannot therefore also to exclude a risk of misclassifying sedentary time as non-wear in adolescent. Our sample size is too small to perform analyses according to age. Future studies should categorize analyses with age as a primary focus.

## Conclusions

Although the concordance was good for most algorithms, the choice of different non-wear time definitions may affect the quantification of the time and PA intensity levels measured, especially sedentary time, using triaxial accelerometry in children and adolescents. Our study highlights the importance of choosing the appropriate processing criteria for assessing PA by accelerometry. Based on our results, we suggest that studies performed in children and adolescents use the algorithm of 30 min of continuous zeros to assess the PA levels and wear time. However, further studies are needed to confirm our hypothesis.

## Additional file

Additional file 1: Log diary. (DOC 5580 kb)

## Abbreviations

CCC: concordance correlation coefficient
MVPA: especially moderate-to-vigorous PA
PA: Physical activity
