# Supplementary material for: Comparison and validation of accelerometer wear time and non-wear time algorithms for assessing physical activity levels in children and adolescents
Source: BMC Med Res Methodol. 2019 Apr 2;19:72. doi: 10.1186/s12874-019-0712-1 (PMC6444637; doi:10.1186/s12874-019-0712-1)
Supplement: Supplementary file 1 — Log diary. (DOC 5580 kb) [file 12874_2019_712_MOESM1_ESM.doc]

The GT3X is worn at the lower back. There is no manipulation to be done, the device is automatic.

There are 4 situations for which it's forbidden or impossible to worn the GT3X :

- for the shower
- When you practice sports with risk of falling: fighting, horse riding …
- In sleep
- During a swimming-pool session

This is why we ask you to note each day the beginning time of the recordings = ‘‘Installation time after awakening.’‘

When you remove the device for the shower, for sport, or rest time, please note the time and tick the box of the corresponding activity.

When the day is over and when you go to bed, note the time of the end of the recordings = ‘‘ Withdrawal time ‘‘.

Thanks for your help.

For questions call your study centre

phone number

**Subject ID** : |__|__| |__|__| |__|__|__|


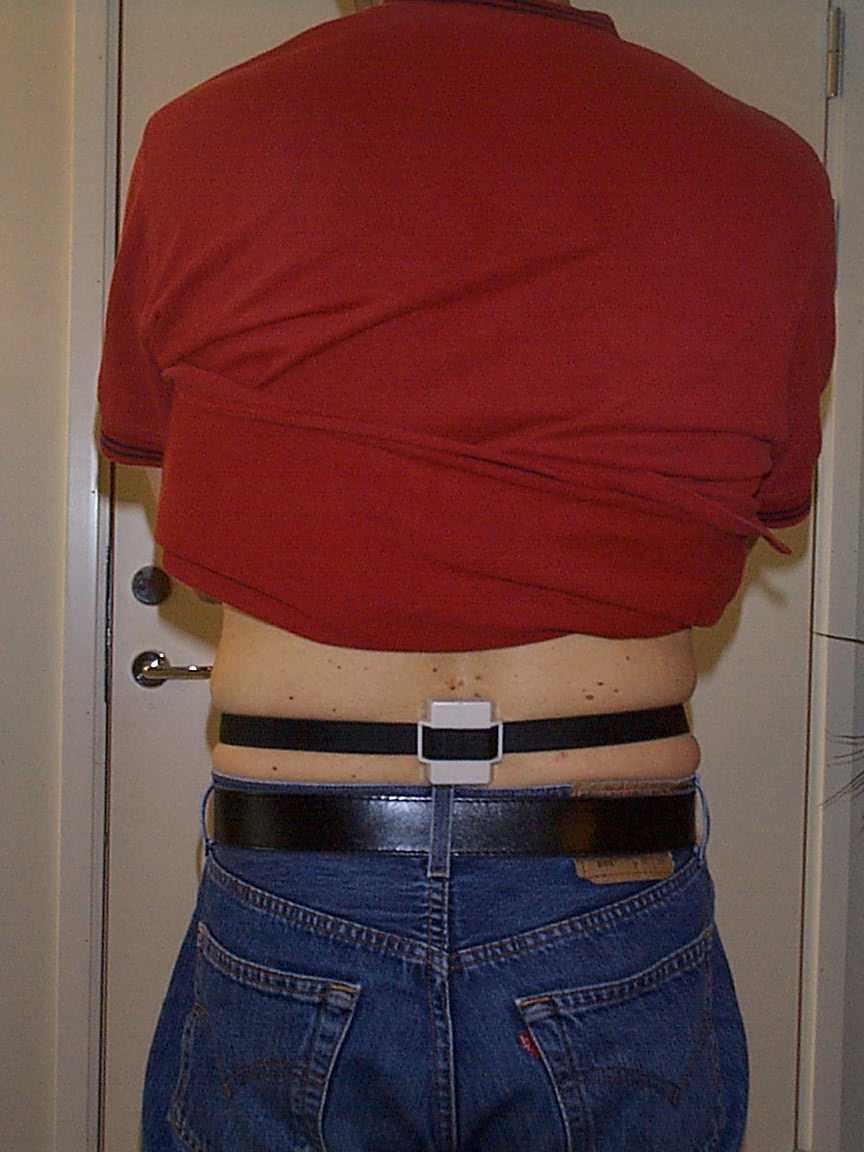


**Day 1:** ………/………/………

**Installation time after awakening**:

……… h ……… min

1. Withdrawal time : ……… h ……… min


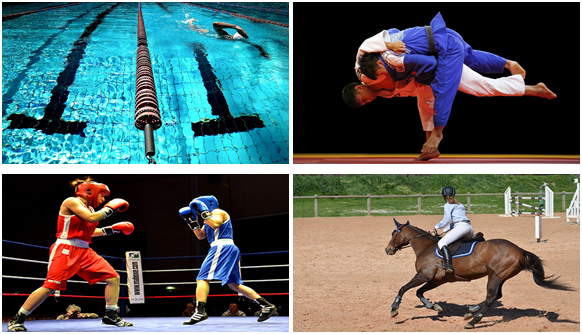


What kind of sport ?

­- Leisure / work

- Club / trainning

- Competition

How long  ?

…….. h ………. min


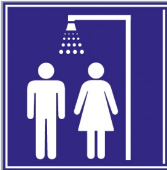


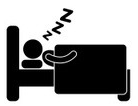


Installation time : ……… h ……… min

2. Withdrawal time: ……… h ……… min


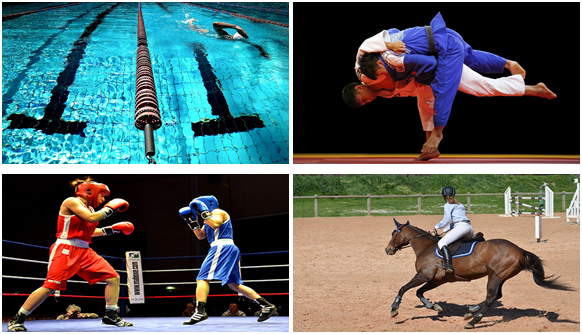


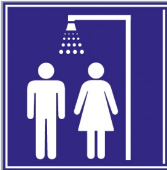


What kind of sport ?

­- Leisure / work

- Club / trainning

- Competition

How long  ?

…….. h ………. min


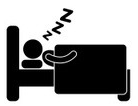


Installation time : ……… h ……… min

3. Withdrawal time: ……… h ……… min


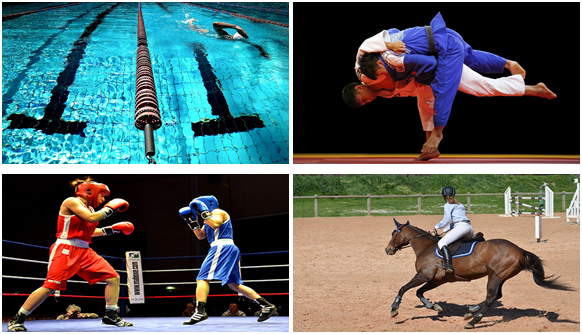


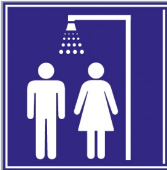


What kind of sport ?

­- Leisure / work

- Club / trainning

- Competition

How long  ?

…….. h ………. min


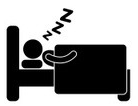


Installation time : ……… h ……… min

**Day 2:** ………/………/………

**Installation time after awakening**:

……… h ……… min

1. Withdrawal time : ……… h ……… min


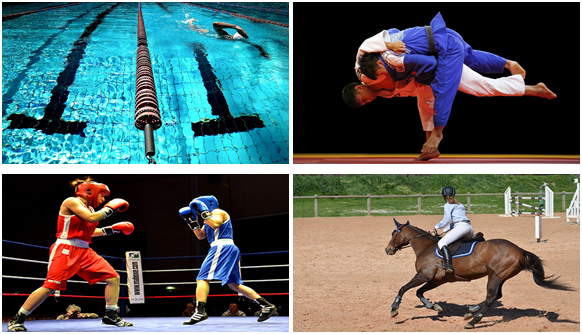


What kind of sport ?

­- Leisure / work

- Club / trainning

- Competition

How long  ?

…….. h ………. min


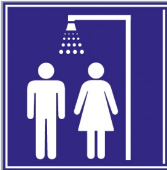


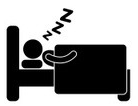


Installation time : ……… h ……… min

2. Withdrawal time: ……… h ……… min


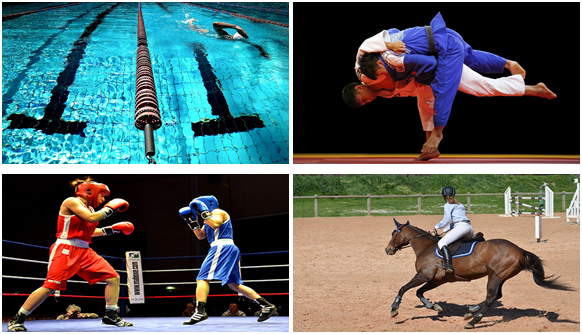


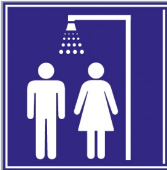


What kind of sport ?

­- Leisure / work

- Club / trainning

- Competition

How long  ?

…….. h ………. min


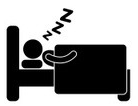


Installation time : ……… h ……… min

3. Withdrawal time: ……… h ……… min


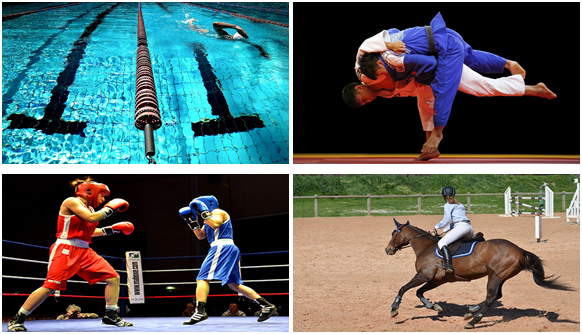


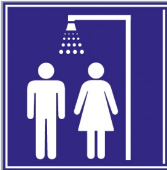


What kind of sport ?

­- Leisure / work

- Club / trainning

- Competition

How long  ?

…….. h ………. min


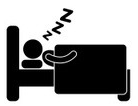


Installation time : ……… h ……… min

**Day 3:** ………/………/………

**Installation time after awakening**:

……… h ……… min

1. Withdrawal time : ……… h ……… min


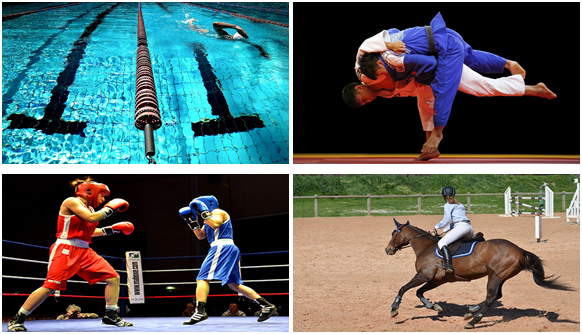


What kind of sport ?

­- Leisure / work

- Club / trainning

- Competition

How long  ?

…….. h ………. min


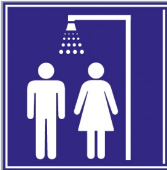


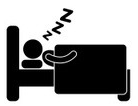


Installation time : ……… h ……… min

2. Withdrawal time: ……… h ……… min


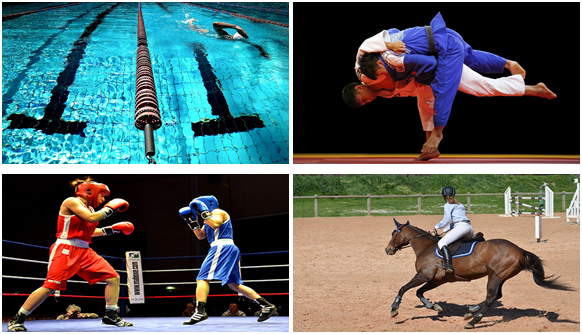


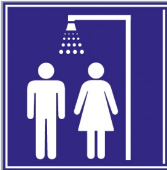


What kind of sport ?

­- Leisure / work

- Club / trainning

- Competition

How long  ?

…….. h ………. min


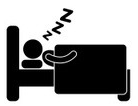


Installation time : ……… h ……… min

3. Withdrawal time: ……… h ……… min


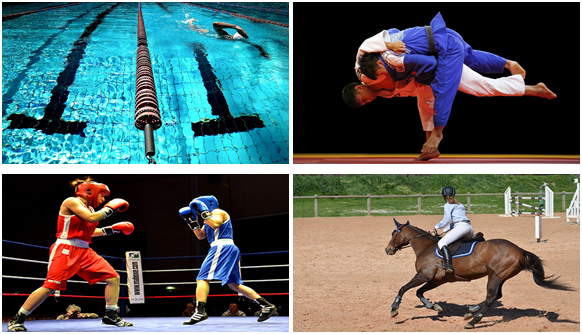


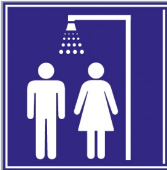


What kind of sport ?

­- Leisure / work

- Club / trainning

- Competition

How long  ?

…….. h ………. min


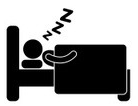


Installation time : ……… h ……… min

**Day 4:** ………/………/………

**Installation time after awakening**:

……… h ……… min

1. Withdrawal time : ……… h ……… min


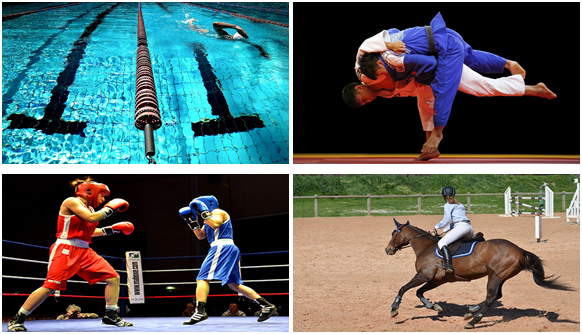


What kind of sport ?

­- Leisure / work

- Club / trainning

- Competition

How long  ?

…….. h ………. min


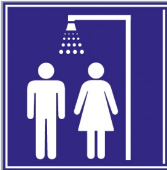


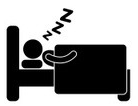


Installation time : ……… h ……… min

2. Withdrawal time: ……… h ……… min


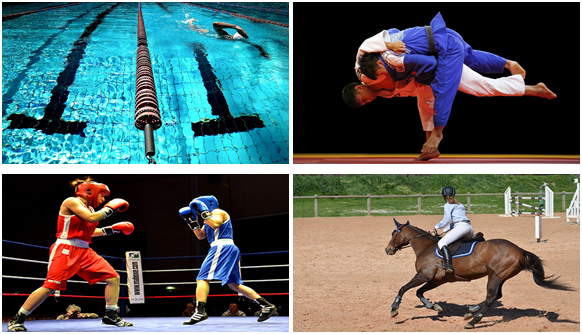


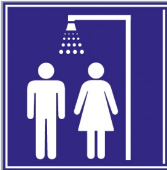


What kind of sport ?

­- Leisure / work

- Club / trainning

- Competition

How long  ?

…….. h ………. min


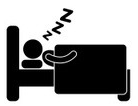


Installation time : ……… h ……… min

3. Withdrawal time: ……… h ……… min


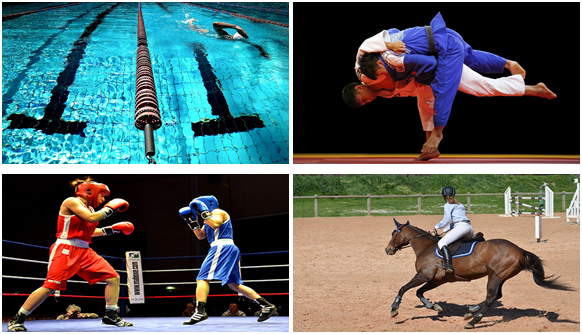


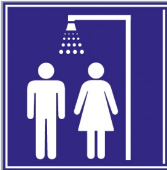


What kind of sport ?

­- Leisure / work

- Club / trainning

- Competition

How long  ?

…….. h ………. min


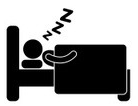


Installation time : ……… h ……… min
